# 2-(4-*tert*-Butyl­phen­yl)-5-{3,4-dibutoxy-5-[5-(4-*tert*-butyl­phen­yl)-1,3,4-oxadiazol-2-yl]-2-thienyl}-1,3,4-oxadiazole

**DOI:** 10.1107/S1600536808033953

**Published:** 2008-10-22

**Authors:** Jian-ning Guan, Hai-lin Li, Lu-na Han, Hai-bo Wang

**Affiliations:** a College of Science, Nanjing University of Technology, Xinmofan Road No. 5 Nanjing, Nanjing 210009, People’s Republic of China

## Abstract

In the title compound, C_36_H_44_N_4_O_4_S, the dihedral angles between the central thio­phene ring and the pendent oxadiazole rings are 12.7 (2) and 13.7 (2)°, and the dihedral angles between the oxadiazole rings and their adjacent benzene rings are 6.1 (2) and 17.5 (2)°. An intra­molecular C—H⋯O inter­action may help to establish the conformation.

## Related literature

For background, see: Laurent *et al.* (2005 ▶).
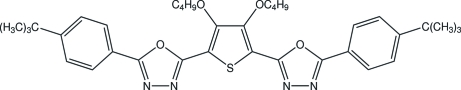


## Experimental

### 

#### Crystal data

C_36_H_44_N_4_O_4_S
*M* *_r_* = 628.81
Monoclinic, 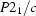

*a* = 19.421 (4) Å
*b* = 17.387 (4) Å
*c* = 10.424 (2) Å
β = 94.24 (3)°
*V* = 3510.3 (12) Å^3^
*Z* = 4
Mo *K*α radiation
μ = 0.14 mm^−1^
*T* = 293 (2) K
0.20 × 0.10 × 0.10 mm

#### Data collection

Enraf–Nonius CAD-4 diffractometer
Absorption correction: ψ scan (North *et al.*, 1968 ▶) *T*_min_ = 0.974, *T*_max_ = 0.987
6675 measured reflections
6295 independent reflections
2847 reflections with *I* > 2σ(*I*)
*R*_int_ = 0.032
3 standard reflections every 200 reflections intensity decay: none

#### Refinement

*R*[*F* ^2^ > 2σ(*F* ^2^)] = 0.085
*wR*(*F* ^2^) = 0.164
*S* = 1.06
6295 reflections
391 parameters
138 restraints
H-atom parameters constrained
Δρ_max_ = 0.17 e Å^−3^
Δρ_min_ = −0.21 e Å^−3^

### 

Data collection: *CAD-4 Software* (Enraf–Nonius, 1989 ▶); cell refinement: *CAD-4 Software*; data reduction: *XCAD4* (Harms & Wocadlo, 1995 ▶); program(s) used to solve structure: *SHELXS97* (Sheldrick, 2008 ▶); program(s) used to refine structure: *SHELXL97* (Sheldrick, 2008 ▶); molecular graphics: *SHELXTL* (Sheldrick, 2008 ▶); software used to prepare material for publication: *SHELXS97*.

## Supplementary Material

Crystal structure: contains datablocks global, I. DOI: 10.1107/S1600536808033953/hb2818sup1.cif

Structure factors: contains datablocks I. DOI: 10.1107/S1600536808033953/hb2818Isup2.hkl

Additional supplementary materials:  crystallographic information; 3D view; checkCIF report

## Figures and Tables

**Table 1:** Hydrogen-bond geometry (Å, °)

*D*—H⋯*A*	*D*—H	H⋯*A*	*D*⋯*A*	*D*—H⋯*A*
C36—H36*B*⋯O2	0.97	2.50	3.149 (6)	124

**Fig. 1.**
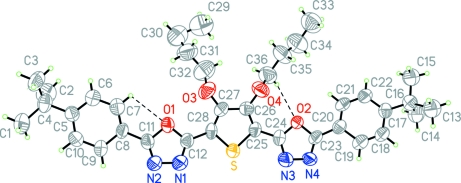
The molecular structure of (I) with displacement ellipsoids for the non-hydrogen atoms drawn at the 30% probability level.

## supplementary crystallographic information

### Comment

Some thiophene derivatives possess biological properties (Laurent *et al.*, 2005). As part of our studies in this area, we report here the synthesis and crystal structure of the title compound, (I).

An intramolecular C—H···O hydrogen bond (Table 1) may help to establish the molecular conformation of (I). The molecular structure of (I) is shown in Fig. 1 and relevant dihedral angles are given in the Abstract.

### Experimental

3,4-Dibutoxythiophene-2,5-dicarbohydrazide (10 mmol) was dissolved in pyridine (30 ml) and 4-*tert*-butylbenzoyl chloride (22 mmol) was added dropwise to the mixture. The resulting mixture was heated to 342 K for 12 h. After cooling, the mixture was poured onto ice water and a white solid was obtained.

The crude compound was dissolved in phosphoryl trichloride (30 ml) and the mixture was refluxed for 12 h. After cooling, the mixture was poured onto crushed ice. The title compound was obtained and purified by recrystalization from trichloromethane. Yield is 85% and melting point is 421 K. Yellow blocks of (I) suitable were obtained by slow evaporation of an ethyl acetate solution.

### Refinement

All H atoms were placed geometrically (C—H = 0.93–0.96 Å) and refined as riding with *U*_iso_(H) = 1.2*U*_eq_(carrier) or 1.5*U*_eq_(methyl carrier).

### Crystal data

**Table:**

C_36_H_44_N_4_O_4_S	*F*(000) = 1344
*M**_r_* = 628.81	*D*_x_ = 1.190 Mg m^−^^3^
Monoclinic, *P*2_1_/*c*	Melting point: 421 K
Hall symbol: -P 2ybc	Mo *K*α radiation, λ = 0.71073 Å
*a* = 19.421 (4) Å	Cell parameters from 25 reflections
*b* = 17.387 (4) Å	θ = 9–12°
*c* = 10.424 (2) Å	µ = 0.14 mm^−^^1^
β = 94.24 (3)°	*T* = 293 K
*V* = 3510.3 (12) Å^3^	Block, yellow
*Z* = 4	0.20 × 0.10 × 0.10 mm

### Data collection

**Table:**

Enraf–Nonius CAD-4 diffractometer	2847 reflections with *I* > 2σ(*I*)
Radiation source: fine-focus sealed tube	*R*_int_ = 0.032
graphite	θ_max_ = 25.2°, θ_min_ = 1.1°
ω/2θ scans	*h* = −23→23
Absorption correction: ψ scan (North *et al.*, 1968)	*k* = 0→20
*T*_min_ = 0.974, *T*_max_ = 0.987	*l* = 0→12
6675 measured reflections	3 standard reflections every 200 reflections
6295 independent reflections	intensity decay: none

### Refinement

**Table:**

Refinement on *F*^2^	Primary atom site location: structure-invariant direct methods
Least-squares matrix: full	Secondary atom site location: difference Fourier map
*R*[*F*^2^ > 2σ(*F*^2^)] = 0.085	Hydrogen site location: inferred from neighbouring sites
*wR*(*F*^2^) = 0.164	H-atom parameters constrained
*S* = 1.06	*w* = 1/[σ^2^(*F*_o_^2^) + (0.03*P*)^2^ + 3*P*] where *P* = (*F*_o_^2^ + 2*F*_c_^2^)/3
6295 reflections	(Δ/σ)_max_ < 0.001
391 parameters	Δρ_max_ = 0.17 e Å^−^^3^
138 restraints	Δρ_min_ = −0.21 e Å^−^^3^

### Special details

**Table:**

Geometry. All e.s.d.'s (except the e.s.d. in the dihedral angle between two l.s. planes) are estimated using the full covariance matrix. The cell e.s.d.'s are taken into account individually in the estimation of e.s.d.'s in distances, angles and torsion angles; correlations between e.s.d.'s in cell parameters are only used when they are defined by crystal symmetry. An approximate (isotropic) treatment of cell e.s.d.'s is used for estimating e.s.d.'s involving l.s. planes.
Refinement. Refinement of *F*^2^ against ALL reflections. The weighted *R*-factor *wR* and goodness of fit *S* are based on *F*^2^, conventional *R*-factors *R* are based on *F*, with *F* set to zero for negative *F*^2^. The threshold expression of *F*^2^ > σ(*F*^2^) is used only for calculating *R*-factors(gt) *etc*. and is not relevant to the choice of reflections for refinement. *R*-factors based on *F*^2^ are statistically about twice as large as those based on *F*, and *R*- factors based on ALL data will be even larger.

### Fractional atomic coordinates and isotropic or equivalent isotropic displacement parameters (Å^2^)

**Table:**

	*x*	*y*	*z*	*U*_iso_*/*U*_eq_	
S	0.21507 (6)	0.13771 (7)	0.42321 (11)	0.0737 (4)	
O1	0.10436 (14)	0.25970 (16)	0.6562 (2)	0.0669 (8)	
O2	0.36725 (13)	0.19395 (15)	0.2071 (2)	0.0642 (8)	
O3	0.21365 (18)	0.3469 (2)	0.5488 (3)	0.0970 (11)	
O4	0.32043 (16)	0.32198 (19)	0.3699 (3)	0.0894 (10)	
N1	0.1172 (2)	0.1352 (2)	0.6394 (4)	0.0923 (12)	
N2	0.0679 (2)	0.1487 (2)	0.7245 (4)	0.0867 (12)	
N3	0.3300 (2)	0.0804 (2)	0.2671 (4)	0.0836 (12)	
N4	0.3820 (2)	0.0680 (2)	0.1858 (4)	0.0888 (13)	
C1	−0.15814 (18)	0.3412 (2)	1.1340 (4)	0.1110 (19)	
H1B	−0.1926	0.3703	1.1743	0.167*	
H1C	−0.1222	0.3265	1.1974	0.167*	
H1D	−0.1788	0.2959	1.0951	0.167*	
C2	−0.18704 (18)	0.4193 (2)	0.9408 (4)	0.122 (2)	
H2B	−0.2195	0.4461	0.9898	0.183*	
H2C	−0.2096	0.3766	0.8970	0.183*	
H2D	−0.1696	0.4538	0.8789	0.183*	
C3	−0.0929 (2)	0.4586 (3)	1.0956 (5)	0.1034 (18)	
H3B	−0.1263	0.4876	1.1389	0.155*	
H3C	−0.0733	0.4905	1.0326	0.155*	
H3D	−0.0571	0.4412	1.1571	0.155*	
C4	−0.1275 (2)	0.3906 (3)	1.0304 (5)	0.0951 (17)	
C5	−0.0756 (2)	0.3445 (3)	0.9573 (4)	0.0738 (13)	
C6	−0.0359 (2)	0.3824 (3)	0.8756 (4)	0.0907 (15)	
H6A	−0.0387	0.4357	0.8684	0.109*	
C7	0.0083 (2)	0.3422 (3)	0.8037 (4)	0.0863 (14)	
H7A	0.0339	0.3694	0.7469	0.104*	
C8	0.0163 (2)	0.2667 (3)	0.8112 (4)	0.0670 (12)	
C9	−0.0241 (2)	0.2255 (3)	0.8991 (4)	0.0895 (15)	
H9A	−0.0211	0.1724	0.9071	0.107*	
C10	−0.0676 (3)	0.2678 (3)	0.9712 (4)	0.0862 (14)	
H10A	−0.0922	0.2424	1.0315	0.103*	
C11	0.0613 (2)	0.2196 (3)	0.7351 (4)	0.0669 (12)	
C12	0.1359 (2)	0.1992 (3)	0.5977 (4)	0.0680 (12)	
C13	0.6959 (3)	0.1763 (3)	−0.0922 (5)	0.1127 (19)	
H13A	0.7334	0.1850	−0.1454	0.169*	
H13B	0.6959	0.1233	−0.0659	0.169*	
H13C	0.7013	0.2087	−0.0176	0.169*	
C14	0.62126 (18)	0.1443 (2)	−0.2875 (3)	0.1019 (17)	
H14A	0.6589	0.1547	−0.3397	0.153*	
H14B	0.5783	0.1550	−0.3359	0.153*	
H14C	0.6224	0.0912	−0.2623	0.153*	
C15	0.63464 (18)	0.2763 (2)	−0.2131 (3)	0.1153 (19)	
H15A	0.6726	0.2798	−0.2668	0.173*	
H15B	0.6429	0.3095	−0.1401	0.173*	
H15C	0.5928	0.2916	−0.2613	0.173*	
C16	0.6278 (2)	0.1950 (3)	−0.1681 (4)	0.0737 (13)	
C17	0.5690 (2)	0.1827 (3)	−0.0836 (4)	0.0673 (12)	
C18	0.5604 (2)	0.1117 (3)	−0.0274 (4)	0.0725 (13)	
H18A	0.5914	0.0725	−0.0422	0.087*	
C19	0.5093 (2)	0.0977 (3)	0.0467 (4)	0.0779 (13)	
H19A	0.5057	0.0493	0.0834	0.093*	
C20	0.4595 (2)	0.1557 (3)	0.0719 (4)	0.0649 (11)	
C21	0.4679 (2)	0.2243 (3)	0.0192 (4)	0.0732 (13)	
H21A	0.4371	0.2635	0.0357	0.088*	
C22	0.5210 (2)	0.2397 (3)	−0.0600 (4)	0.0735 (12)	
H22A	0.5244	0.2880	−0.0972	0.088*	
C23	0.4030 (2)	0.1344 (3)	0.1547 (4)	0.0662 (12)	
C24	0.3240 (2)	0.1546 (3)	0.2765 (4)	0.0656 (12)	
C25	0.2752 (2)	0.1947 (3)	0.3536 (4)	0.0642 (11)	
C26	0.2736 (2)	0.2678 (3)	0.3940 (4)	0.0637 (11)	
C27	0.2228 (2)	0.2800 (3)	0.4822 (4)	0.0711 (13)	
C28	0.1870 (2)	0.2153 (3)	0.5084 (4)	0.0668 (12)	
C29	0.1698 (4)	0.5589 (4)	0.4059 (7)	0.163 (3)	
H29A	0.1491	0.6006	0.3566	0.245*	
H29B	0.1763	0.5159	0.3502	0.245*	
H29C	0.2137	0.5749	0.4454	0.245*	
C30	0.1233 (4)	0.5356 (4)	0.5085 (7)	0.153 (3)	
H30A	0.1163	0.5762	0.5700	0.184*	
H30B	0.0793	0.5151	0.4740	0.184*	
C31	0.1722 (3)	0.4753 (3)	0.5594 (6)	0.123 (2)	
H31A	0.2154	0.5013	0.5839	0.148*	
H31B	0.1547	0.4566	0.6383	0.148*	
C32	0.1891 (3)	0.4099 (4)	0.4886 (6)	0.129 (2)	
H32A	0.1479	0.3950	0.4364	0.155*	
H32B	0.2229	0.4258	0.4298	0.155*	
C33	0.4367 (3)	0.5060 (4)	0.1190 (6)	0.146 (2)	
H33A	0.4471	0.5140	0.0314	0.218*	
H33B	0.4774	0.4888	0.1686	0.218*	
H33C	0.4211	0.5534	0.1541	0.218*	
C34	0.3837 (3)	0.4491 (4)	0.1234 (6)	0.137 (2)	
H34A	0.3964	0.4057	0.0715	0.165*	
H34B	0.3415	0.4705	0.0826	0.165*	
C35	0.3683 (3)	0.4197 (3)	0.2492 (5)	0.1124 (19)	
H35A	0.3565	0.4634	0.3011	0.135*	
H35B	0.4107	0.3981	0.2893	0.135*	
C36	0.3132 (3)	0.3612 (3)	0.2580 (5)	0.1072 (18)	
H36A	0.2685	0.3865	0.2510	0.129*	
H36B	0.3146	0.3254	0.1868	0.129*	

### Atomic displacement parameters (Å^2^)

**Table:**

	*U*^11^	*U*^22^	*U*^33^	*U*^12^	*U*^13^	*U*^23^
S	0.0887 (8)	0.0642 (7)	0.0711 (7)	−0.0006 (7)	0.0253 (6)	−0.0056 (7)
O1	0.0741 (18)	0.0740 (19)	0.0540 (16)	0.0001 (15)	0.0148 (14)	−0.0039 (15)
O2	0.0681 (17)	0.0533 (17)	0.0740 (19)	0.0009 (15)	0.0247 (15)	−0.0071 (15)
O3	0.130 (3)	0.079 (3)	0.083 (2)	0.008 (2)	0.021 (2)	−0.028 (2)
O4	0.097 (2)	0.082 (2)	0.092 (2)	−0.028 (2)	0.017 (2)	−0.004 (2)
N1	0.126 (3)	0.066 (3)	0.088 (3)	0.006 (2)	0.033 (2)	−0.008 (2)
N2	0.118 (3)	0.063 (2)	0.082 (3)	0.000 (2)	0.033 (2)	−0.012 (2)
N3	0.094 (3)	0.070 (3)	0.089 (3)	−0.006 (2)	0.024 (2)	−0.005 (2)
N4	0.120 (3)	0.056 (3)	0.098 (3)	0.001 (2)	0.056 (3)	0.001 (2)
C1	0.108 (4)	0.121 (5)	0.110 (4)	0.015 (4)	0.045 (3)	−0.007 (4)
C2	0.100 (4)	0.136 (5)	0.127 (5)	0.019 (4)	−0.010 (4)	−0.001 (4)
C3	0.084 (3)	0.098 (4)	0.133 (5)	0.003 (3)	0.038 (3)	−0.037 (4)
C4	0.062 (3)	0.105 (4)	0.124 (4)	0.023 (3)	0.046 (3)	−0.004 (4)
C5	0.082 (3)	0.071 (3)	0.070 (3)	−0.010 (3)	0.018 (2)	−0.005 (3)
C6	0.102 (4)	0.079 (3)	0.095 (3)	0.000 (3)	0.031 (3)	−0.005 (3)
C7	0.090 (3)	0.088 (3)	0.082 (3)	0.006 (3)	0.021 (3)	0.004 (3)
C8	0.072 (3)	0.070 (3)	0.062 (3)	0.001 (2)	0.020 (2)	−0.016 (2)
C9	0.112 (4)	0.081 (3)	0.082 (3)	0.014 (3)	0.045 (3)	0.003 (3)
C10	0.114 (4)	0.071 (3)	0.077 (3)	−0.012 (3)	0.032 (3)	0.001 (3)
C11	0.068 (3)	0.077 (3)	0.056 (3)	0.005 (2)	0.010 (2)	0.002 (2)
C12	0.077 (3)	0.074 (3)	0.054 (3)	0.011 (3)	0.011 (2)	0.001 (2)
C13	0.094 (4)	0.140 (5)	0.105 (4)	0.006 (4)	0.016 (3)	0.009 (4)
C14	0.120 (4)	0.108 (4)	0.083 (4)	0.004 (3)	0.041 (3)	−0.008 (3)
C15	0.109 (4)	0.117 (5)	0.121 (5)	−0.010 (4)	0.011 (4)	0.003 (4)
C16	0.083 (3)	0.083 (3)	0.056 (3)	−0.001 (3)	0.014 (2)	−0.002 (3)
C17	0.066 (3)	0.076 (3)	0.060 (3)	0.001 (2)	0.007 (2)	−0.008 (2)
C18	0.080 (3)	0.068 (3)	0.070 (3)	0.018 (2)	0.009 (2)	−0.001 (2)
C19	0.094 (3)	0.073 (3)	0.067 (3)	0.018 (3)	0.006 (3)	−0.001 (2)
C20	0.067 (3)	0.066 (3)	0.062 (3)	0.008 (2)	0.000 (2)	−0.010 (2)
C21	0.086 (3)	0.057 (3)	0.077 (3)	0.011 (2)	0.009 (2)	−0.015 (2)
C22	0.075 (3)	0.067 (3)	0.078 (3)	0.006 (2)	0.007 (2)	−0.005 (2)
C23	0.079 (3)	0.061 (3)	0.059 (3)	0.020 (3)	0.011 (2)	−0.003 (3)
C24	0.056 (3)	0.079 (3)	0.062 (3)	0.005 (2)	0.008 (2)	−0.008 (3)
C25	0.061 (3)	0.074 (3)	0.059 (3)	0.002 (2)	0.012 (2)	−0.011 (2)
C26	0.070 (3)	0.060 (3)	0.060 (3)	−0.003 (2)	0.001 (2)	−0.005 (2)
C27	0.080 (3)	0.061 (3)	0.073 (3)	0.005 (3)	0.014 (3)	−0.015 (3)
C28	0.071 (3)	0.084 (3)	0.047 (2)	0.004 (3)	0.020 (2)	−0.012 (2)
C29	0.181 (7)	0.152 (7)	0.153 (7)	0.007 (6)	−0.013 (6)	0.002 (5)
C30	0.171 (7)	0.133 (6)	0.159 (7)	0.013 (6)	0.029 (6)	−0.011 (5)
C31	0.160 (6)	0.084 (4)	0.126 (5)	0.014 (4)	0.016 (5)	0.006 (4)
C32	0.138 (6)	0.119 (6)	0.127 (6)	0.032 (5)	−0.015 (4)	0.000 (5)
C33	0.147 (6)	0.143 (6)	0.149 (6)	−0.011 (5)	0.023 (5)	0.027 (5)
C34	0.128 (6)	0.140 (6)	0.143 (6)	−0.023 (5)	−0.002 (5)	0.009 (5)
C35	0.095 (4)	0.119 (5)	0.123 (5)	−0.027 (4)	0.008 (4)	0.029 (4)
C36	0.106 (4)	0.105 (4)	0.111 (5)	0.003 (4)	0.006 (4)	0.024 (4)

### Geometric parameters (Å, °)

**Table:**

S—C28	1.726 (4)	C14—H14B	0.9600
S—C25	1.732 (4)	C14—H14C	0.9600
O1—C12	1.382 (5)	C15—C16	1.499 (5)
O1—C11	1.402 (4)	C15—H15A	0.9600
O2—C24	1.336 (4)	C15—H15B	0.9600
O2—C23	1.380 (4)	C15—H15C	0.9600
O3—C32	1.334 (6)	C16—C17	1.507 (5)
O3—C27	1.374 (5)	C17—C18	1.383 (5)
O4—C26	1.345 (5)	C17—C22	1.394 (5)
O4—C36	1.349 (5)	C18—C19	1.324 (5)
N1—C12	1.259 (5)	C18—H18A	0.9300
N1—N2	1.372 (5)	C19—C20	1.434 (5)
N2—C11	1.245 (5)	C19—H19A	0.9300
N3—C24	1.300 (5)	C20—C21	1.329 (5)
N3—N4	1.383 (4)	C20—C23	1.493 (5)
N4—C23	1.275 (5)	C21—C22	1.394 (5)
C1—C4	1.533 (6)	C21—H21A	0.9300
C1—H1B	0.9600	C22—H22A	0.9300
C1—H1C	0.9600	C24—C25	1.464 (5)
C1—H1D	0.9600	C25—C26	1.340 (5)
C2—C4	1.517 (6)	C26—C27	1.414 (5)
C2—H2B	0.9600	C27—C28	1.359 (5)
C2—H2C	0.9599	C29—C30	1.506 (8)
C2—H2D	0.9600	C29—H29A	0.9600
C3—C4	1.497 (6)	C29—H29B	0.9600
C3—H3B	0.9600	C29—H29C	0.9600
C3—H3C	0.9600	C30—C31	1.487 (8)
C3—H3D	0.9600	C30—H30A	0.9700
C4—C5	1.533 (6)	C30—H30B	0.9700
C5—C10	1.350 (6)	C31—C32	1.407 (6)
C5—C6	1.361 (5)	C31—H31A	0.9700
C6—C7	1.371 (6)	C31—H31B	0.9700
C6—H6A	0.9300	C32—H32A	0.9700
C7—C8	1.324 (5)	C32—H32B	0.9700
C7—H7A	0.9300	C33—C34	1.432 (7)
C8—C9	1.440 (5)	C33—H33A	0.9600
C8—C11	1.471 (5)	C33—H33B	0.9600
C9—C10	1.382 (5)	C33—H33C	0.9600
C9—H9A	0.9300	C34—C35	1.458 (7)
C10—H10A	0.9300	C34—H34A	0.9700
C12—C28	1.438 (5)	C34—H34B	0.9700
C13—C16	1.526 (6)	C35—C36	1.485 (6)
C13—H13A	0.9600	C35—H35A	0.9700
C13—H13B	0.9600	C35—H35B	0.9700
C13—H13C	0.9600	C36—H36A	0.9700
C14—C16	1.523 (5)	C36—H36B	0.9700
C14—H14A	0.9600		
			
C28—S—C25	90.9 (2)	C22—C17—C16	123.2 (4)
C12—O1—C11	100.6 (3)	C19—C18—C17	122.0 (4)
C24—O2—C23	100.6 (3)	C19—C18—H18A	119.0
C32—O3—C27	120.9 (4)	C17—C18—H18A	119.0
C26—O4—C36	119.1 (4)	C18—C19—C20	121.5 (4)
C12—N1—N2	107.8 (4)	C18—C19—H19A	119.2
C11—N2—N1	108.0 (4)	C20—C19—H19A	119.2
C24—N3—N4	105.9 (3)	C21—C20—C19	116.8 (4)
C23—N4—N3	106.0 (3)	C21—C20—C23	125.3 (4)
C4—C1—H1B	109.5	C19—C20—C23	117.8 (4)
C4—C1—H1C	109.5	C20—C21—C22	122.3 (4)
H1B—C1—H1C	109.5	C20—C21—H21A	118.9
C4—C1—H1D	109.5	C22—C21—H21A	118.9
H1B—C1—H1D	109.5	C17—C22—C21	120.2 (4)
H1C—C1—H1D	109.5	C17—C22—H22A	119.9
C4—C2—H2B	109.5	C21—C22—H22A	119.9
C4—C2—H2C	109.8	N4—C23—O2	113.6 (4)
H2B—C2—H2C	109.5	N4—C23—C20	129.3 (4)
C4—C2—H2D	109.2	O2—C23—C20	117.1 (4)
H2B—C2—H2D	109.5	N3—C24—O2	113.8 (3)
H2C—C2—H2D	109.5	N3—C24—C25	125.4 (4)
C4—C3—H3B	109.5	O2—C24—C25	120.8 (4)
C4—C3—H3C	109.5	C26—C25—C24	131.0 (4)
H3B—C3—H3C	109.5	C26—C25—S	112.2 (3)
C4—C3—H3D	109.5	C24—C25—S	116.2 (3)
H3B—C3—H3D	109.5	C25—C26—O4	124.9 (4)
H3C—C3—H3D	109.5	C25—C26—C27	112.3 (4)
C3—C4—C2	108.3 (4)	O4—C26—C27	122.1 (4)
C3—C4—C5	110.4 (4)	C28—C27—O3	120.8 (4)
C2—C4—C5	111.4 (4)	C28—C27—C26	113.5 (4)
C3—C4—C1	107.9 (4)	O3—C27—C26	125.3 (4)
C2—C4—C1	107.5 (3)	C27—C28—C12	132.8 (4)
C5—C4—C1	111.2 (4)	C27—C28—S	111.0 (3)
C10—C5—C6	118.6 (4)	C12—C28—S	116.1 (3)
C10—C5—C4	122.5 (4)	C30—C29—H29A	109.5
C6—C5—C4	118.8 (4)	C30—C29—H29B	109.5
C5—C6—C7	120.1 (5)	H29A—C29—H29B	109.5
C5—C6—H6A	120.0	C30—C29—H29C	109.5
C7—C6—H6A	120.0	H29A—C29—H29C	109.5
C8—C7—C6	123.3 (5)	H29B—C29—H29C	109.5
C8—C7—H7A	118.3	C31—C30—C29	92.4 (6)
C6—C7—H7A	118.3	C31—C30—H30A	113.2
C7—C8—C9	117.6 (4)	C29—C30—H30A	113.2
C7—C8—C11	126.3 (4)	C31—C30—H30B	113.2
C9—C8—C11	116.1 (4)	C29—C30—H30B	113.2
C10—C9—C8	117.7 (4)	H30A—C30—H30B	110.6
C10—C9—H9A	121.1	C32—C31—C30	123.4 (6)
C8—C9—H9A	121.1	C32—C31—H31A	106.5
C5—C10—C9	122.5 (4)	C30—C31—H31A	106.5
C5—C10—H10A	118.7	C32—C31—H31B	106.5
C9—C10—H10A	118.7	C30—C31—H31B	106.5
N2—C11—O1	111.8 (4)	H31A—C31—H31B	106.5
N2—C11—C8	131.9 (4)	O3—C32—C31	120.4 (6)
O1—C11—C8	116.3 (4)	O3—C32—H32A	107.2
N1—C12—O1	111.8 (4)	C31—C32—H32A	107.2
N1—C12—C28	128.9 (4)	O3—C32—H32B	107.2
O1—C12—C28	119.1 (4)	C31—C32—H32B	107.2
C16—C13—H13A	109.5	H32A—C32—H32B	106.9
C16—C13—H13B	109.5	C34—C33—H33A	109.5
H13A—C13—H13B	109.5	C34—C33—H33B	109.5
C16—C13—H13C	109.5	H33A—C33—H33B	109.5
H13A—C13—H13C	109.5	C34—C33—H33C	109.5
H13B—C13—H13C	109.5	H33A—C33—H33C	109.5
C16—C14—H14A	109.5	H33B—C33—H33C	109.5
C16—C14—H14B	109.5	C33—C34—C35	117.8 (6)
H14A—C14—H14B	109.5	C33—C34—H34A	107.9
C16—C14—H14C	109.5	C35—C34—H34A	107.9
H14A—C14—H14C	109.5	C33—C34—H34B	107.9
H14B—C14—H14C	109.5	C35—C34—H34B	107.9
C16—C15—H15A	109.5	H34A—C34—H34B	107.2
C16—C15—H15B	109.5	C34—C35—C36	119.5 (5)
H15A—C15—H15B	109.5	C34—C35—H35A	107.5
C16—C15—H15C	109.5	C36—C35—H35A	107.5
H15A—C15—H15C	109.5	C34—C35—H35B	107.5
H15B—C15—H15C	109.5	C36—C35—H35B	107.5
C15—C16—C17	113.9 (4)	H35A—C35—H35B	107.0
C15—C16—C14	107.1 (3)	O4—C36—C35	111.7 (5)
C17—C16—C14	112.1 (4)	O4—C36—H36A	109.3
C15—C16—C13	105.6 (4)	C35—C36—H36A	109.3
C17—C16—C13	109.4 (4)	O4—C36—H36B	109.3
C14—C16—C13	108.5 (4)	C35—C36—H36B	109.3
C18—C17—C22	117.1 (4)	H36A—C36—H36B	107.9
C18—C17—C16	119.6 (4)		
			
C28—S—C25—C24	172.5 (3)	C9—C8—C11—N2	6.1 (7)
C28—S—C25—C26	0.4 (3)	C8—C9—C10—C5	3.3 (7)
C25—S—C28—C12	−175.5 (3)	N1—C12—C28—S	13.7 (6)
C25—S—C28—C27	0.1 (3)	O1—C12—C28—S	−171.4 (3)
C12—O1—C11—N2	0.8 (4)	N1—C12—C28—C27	−160.6 (5)
C12—O1—C11—C8	−177.9 (3)	O1—C12—C28—C27	14.3 (7)
C11—O1—C12—N1	−2.8 (4)	C13—C16—C17—C18	−57.0 (6)
C11—O1—C12—C28	−178.6 (4)	C13—C16—C17—C22	124.3 (5)
C24—O2—C23—N4	1.9 (5)	C14—C16—C17—C18	63.2 (5)
C24—O2—C23—C20	−177.8 (4)	C14—C16—C17—C22	−115.5 (5)
C23—O2—C24—N3	−1.5 (4)	C15—C16—C17—C18	−174.9 (4)
C23—O2—C24—C25	178.9 (4)	C15—C16—C17—C22	6.5 (6)
C32—O3—C27—C26	70.3 (6)	C22—C17—C18—C19	−0.6 (6)
C32—O3—C27—C28	−117.5 (5)	C16—C17—C22—C21	179.8 (4)
C27—O3—C32—C31	172.5 (5)	C16—C17—C18—C19	−179.3 (4)
C36—O4—C26—C25	84.4 (6)	C18—C17—C22—C21	1.2 (6)
C36—O4—C26—C27	−105.3 (5)	C17—C18—C19—C20	0.5 (7)
C26—O4—C36—C35	178.6 (4)	C18—C19—C20—C21	−1.0 (6)
N2—N1—C12—O1	3.8 (5)	C18—C19—C20—C23	178.9 (4)
N2—N1—C12—C28	179.0 (4)	C23—C20—C21—C22	−178.3 (4)
C12—N1—N2—C11	−3.2 (5)	C19—C20—C23—O2	162.7 (4)
N1—N2—C11—O1	1.4 (5)	C19—C20—C23—N4	−16.9 (7)
N1—N2—C11—C8	179.7 (4)	C21—C20—C23—O2	−17.4 (6)
C24—N3—N4—C23	0.6 (5)	C19—C20—C21—C22	1.7 (6)
N4—N3—C24—O2	0.7 (5)	C21—C20—C23—N4	163.0 (5)
N4—N3—C24—C25	−179.7 (4)	C20—C21—C22—C17	−1.8 (7)
N3—N4—C23—C20	178.1 (4)	O2—C24—C25—S	172.4 (3)
N3—N4—C23—O2	−1.6 (5)	N3—C24—C25—S	−7.1 (6)
C1—C4—C5—C6	−170.1 (4)	N3—C24—C25—C26	163.2 (5)
C3—C4—C5—C10	129.2 (5)	O2—C24—C25—C26	−17.2 (7)
C3—C4—C5—C6	−50.5 (6)	S—C25—C26—O4	170.4 (3)
C2—C4—C5—C10	−110.3 (5)	C24—C25—C26—C27	−171.4 (4)
C1—C4—C5—C10	9.5 (6)	S—C25—C26—C27	−0.7 (5)
C2—C4—C5—C6	70.0 (5)	C24—C25—C26—O4	−0.2 (8)
C10—C5—C6—C7	3.9 (7)	O4—C26—C27—C28	−170.7 (4)
C4—C5—C10—C9	175.7 (4)	C25—C26—C27—O3	173.5 (4)
C6—C5—C10—C9	−4.7 (7)	C25—C26—C27—C28	0.8 (5)
C4—C5—C6—C7	−176.5 (4)	O4—C26—C27—O3	2.0 (7)
C5—C6—C7—C8	−1.9 (7)	O3—C27—C28—S	−173.5 (3)
C6—C7—C8—C9	0.5 (6)	O3—C27—C28—C12	1.0 (7)
C6—C7—C8—C11	178.3 (4)	C26—C27—C28—S	−0.5 (5)
C9—C8—C11—O1	−175.6 (3)	C26—C27—C28—C12	174.1 (4)
C7—C8—C11—O1	6.6 (6)	C29—C30—C31—C32	−64.9 (7)
C7—C8—C11—N2	−171.8 (5)	C30—C31—C32—O3	−157.7 (6)
C7—C8—C9—C10	−1.1 (6)	C33—C34—C35—C36	179.3 (5)
C11—C8—C9—C10	−179.2 (4)	C34—C35—C36—O4	159.5 (5)

### Hydrogen-bond geometry (Å, °)

**Table:**

*D*—H···*A*	*D*—H	H···*A*	*D*···*A*	*D*—H···*A*
C36—H36B···O2	0.97	2.50	3.149 (6)	124
